# A step by step guide for conducting a systematic review and meta-analysis with simulation data

**DOI:** 10.1186/s41182-019-0165-6

**Published:** 2019-08-01

**Authors:** Gehad Mohamed Tawfik, Kadek Agus Surya Dila, Muawia Yousif Fadlelmola Mohamed, Dao Ngoc Hien Tam, Nguyen Dang Kien, Ali Mahmoud Ahmed, Nguyen Tien Huy

**Affiliations:** 1 Faculty of Medicine, Ain Shams University, Cairo, Egypt; 2 Online research Club, http://www.onlineresearchclub.org/; 3 Pratama Giri Emas Hospital, Singaraja-Amlapura street, Giri Emas village, Sawan subdistrict, Singaraja City, Buleleng, Bali 81171 Indonesia; 4 Faculty of Medicine, University of Khartoum, Khartoum, Sudan; 5 Nanogen Pharmaceutical Biotechnology Joint Stock Company, Ho Chi Minh City, Vietnam; 6 Department of Obstetrics and Gynecology, Thai Binh University of Medicine and Pharmacy, Thai Binh, Vietnam; 7 Faculty of Medicine, Al-Azhar University, Cairo, Egypt; 8 Evidence Based Medicine Research Group & Faculty of Applied Sciences, Ton Duc Thang University, Ho Chi Minh City, 70000 Vietnam; 9 Faculty of Applied Sciences, Ton Duc Thang University, Ho Chi Minh City, 70000 Vietnam; 10 Department of Clinical Product Development, Institute of Tropical Medicine (NEKKEN), Leading Graduate School Program, and Graduate School of Biomedical Sciences, Nagasaki University, 1-12-4 Sakamoto, Nagasaki, 852-8523 Japan

**Keywords:** Search, Data, Extraction, Analysis, Study, Results

## Abstract

**Background:**

The massive abundance of studies relating to tropical medicine and health has increased strikingly over the last few decades. In the field of tropical medicine and health, a well-conducted systematic review and meta-analysis (SR/MA) is considered a feasible solution for keeping clinicians abreast of current evidence-based medicine. Understanding of SR/MA steps is of paramount importance for its conduction. It is not easy to be done as there are obstacles that could face the researcher. To solve those hindrances, this methodology study aimed to provide a step-by-step approach mainly for beginners and junior researchers, in the field of tropical medicine and other health care fields, on how to properly conduct a SR/MA, in which all the steps here depicts our experience and expertise combined with the already well-known and accepted international guidance.

We suggest that all steps of SR/MA should be done independently by 2–3 reviewers’ discussion, to ensure data quality and accuracy.

**Conclusion:**

SR/MA steps include the development of research question, forming criteria, search strategy, searching databases, protocol registration, title, abstract, full-text screening, manual searching, extracting data, quality assessment, data checking, statistical analysis, double data checking, and manuscript writing.

**Electronic supplementary material:**

The online version of this article (10.1186/s41182-019-0165-6) contains supplementary material, which is available to authorized users.

## Introduction

The amount of studies published in the biomedical literature, especially tropical medicine and health, has increased strikingly over the last few decades. This massive abundance of literature makes clinical medicine increasingly complex, and knowledge from various researches is often needed to inform a particular clinical decision. However, available studies are often heterogeneous with regard to their design, operational quality, and subjects under study and may handle the research question in a different way, which adds to the complexity of evidence and conclusion synthesis [1].

Systematic review and meta-analyses (SR/MAs) have a high level of evidence as represented by the evidence-based pyramid. Therefore, a well-conducted SR/MA is considered a feasible solution in keeping health clinicians ahead regarding contemporary evidence-based medicine.

Differing from a systematic review, unsystematic narrative review tends to be descriptive, in which the authors select frequently articles based on their point of view which leads to its poor quality. A systematic review, on the other hand, is defined as a review using a systematic method to summarize evidence on questions with a detailed and comprehensive plan of study. Furthermore, despite the increasing guidelines for effectively conducting a systematic review, we found that basic steps often start from framing question, then identifying relevant work which consists of criteria development and search for articles, appraise the quality of included studies, summarize the evidence, and interpret the results [2, 3]. However, those simple steps are not easy to be reached in reality. There are many troubles that a researcher could be struggled with which has no detailed indication.

**Fig. 1:**
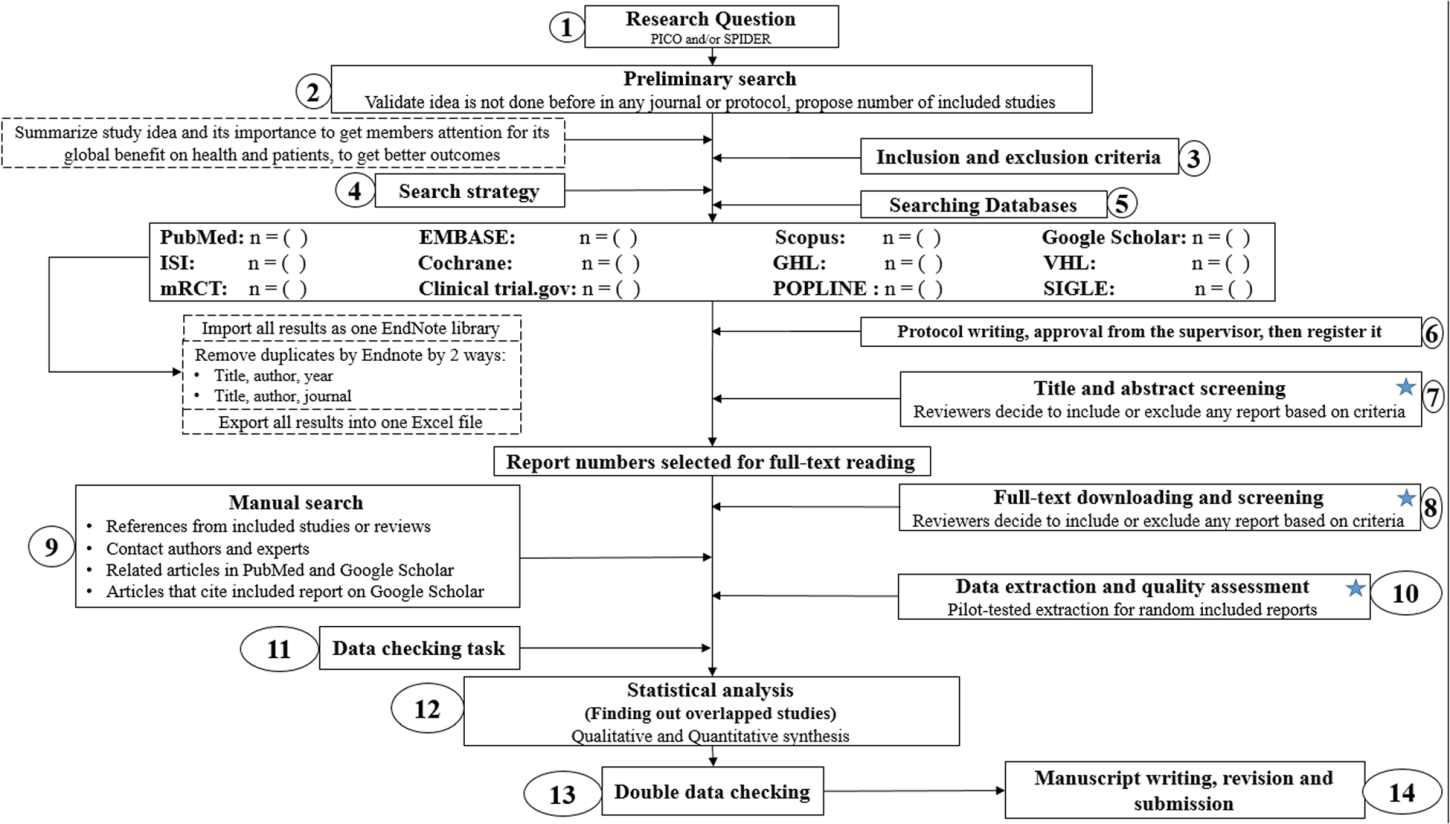
Detailed flow diagram guideline for systematic review and meta-analysis steps. *Note*: Star icon refers to “2–3 reviewers screen independently”

Conducting a SR/MA in tropical medicine and health may be difficult especially for young researchers; therefore, understanding of its essential steps is crucial. It is not easy to be done as there are obstacles that could face the researcher. To solve those hindrances, we recommend a flow diagram (Fig. 1) which illustrates a detailed and step-by-step the stages for SR/MA studies. This methodology study aimed to provide a step-by-step approach mainly for beginners and junior researchers, in the field of tropical medicine and other health care fields, on how to properly and succinctly conduct a SR/MA; all the steps here depicts our experience and expertise combined with the already well known and accepted international guidance.

## Methods and results

### Detailed steps for conducting any systematic review and meta-analysis

We searched the methods reported in published SR/MA in tropical medicine and other healthcare fields besides the published guidelines like Cochrane guidelines {Higgins, 2011 #7} [4] to collect the best low-bias method for each step of SR/MA conduction steps. Furthermore, we used guidelines that we apply in studies for all SR/MA steps. We combined these methods in order to conclude and conduct a detailed flow diagram that shows the SR/MA steps how being conducted.

Any SR/MA must follow the widely accepted Preferred Reporting Items for Systematic Review and Meta-analysis statement (PRISMA checklist 2009) (Additional file 5: Table S1) [5].

We proposed our methods according to a valid explanatory simulation example choosing the topic of “evaluating safety of Ebola vaccine,” as it is known that Ebola is a very rare tropical disease but fatal. All the explained methods feature the standards followed internationally, with our compiled experience in the conduct of SR beside it, which we think proved some validity. This is a SR under conduct by a couple of researchers teaming in a research group, moreover, as the outbreak of Ebola which took place (2013–2016) in Africa resulted in a significant mortality and morbidity. Furthermore, since there are many published and ongoing trials assessing the safety of Ebola vaccines, we thought this would provide a great opportunity to tackle this hotly debated issue. Moreover, Ebola started to fire again and new fatal outbreak appeared in the Democratic Republic of Congo since August 2018, which caused infection to more than 1000 people according to the World Health Organization, and 629 people have been killed till now. Hence, it is considered the second worst Ebola outbreak, after the first one in West Africa in 2014, which infected more than 26,000 and killed about 11,300 people along outbreak course.

#### Research question and objectives

Like other study designs, the research question of SR/MA should be feasible, interesting, novel, ethical, and relevant. Therefore, a clear, logical, and well-defined research question should be formulated. Usually, two common tools are used: PICO or SPIDER. PICO (Population, Intervention, Comparison, Outcome) is used mostly in quantitative evidence synthesis. Authors demonstrated that PICO holds more sensitivity than the more specific SPIDER approach [6]. SPIDER (Sample, Phenomenon of Interest, Design, Evaluation, Research type) was proposed as a method for qualitative and mixed methods search.

We here recommend a combined approach of using either one or both the SPIDER and PICO tools to retrieve a comprehensive search depending on time and resources limitations. When we apply this to our assumed research topic, being of qualitative nature, the use of SPIDER approach is more valid.

PICO is usually used for systematic review and meta-analysis of clinical trial study. For the observational study (without intervention or comparator), in many tropical and epidemiological questions, it is usually enough to use P (Patient) and O (outcome) only to formulate a research question. We must indicate clearly the population (P), then intervention (I) or exposure. Next, it is necessary to compare (C) the indicated intervention with other interventions, i.e., placebo. Finally, we need to clarify which are our relevant outcomes.

To facilitate comprehension, we choose the Ebola virus disease (EVD) as an example. Currently, the vaccine for EVD is being developed and under phase I, II, and III clinical trials; we want to know whether this vaccine is safe and can induce sufficient immunogenicity to the subjects.

An example of a research question for SR/MA based on PICO for this issue is as follows: How is the safety and immunogenicity of Ebola vaccine in human? (P: healthy subjects (human), I: vaccination, C: placebo, O: safety or adverse effects)

#### Preliminary research and idea validation

We recommend a preliminary search to identify relevant articles, ensure the validity of the proposed idea, avoid duplication of previously addressed questions, and assure that we have enough articles for conducting its analysis. Moreover, themes should focus on relevant and important health-care issues, consider global needs and values, reflect the current science, and be consistent with the adopted review methods. Gaining familiarity with a deep understanding of the study field through relevant videos and discussions is of paramount importance for better retrieval of results. If we ignore this step, our study could be canceled whenever we find out a similar study published before. This means we are wasting our time to deal with a problem that has been tackled for a long time.

To do this, we can start by doing a simple search in PubMed or Google Scholar with search terms Ebola AND vaccine. While doing this step, we identify a systematic review and meta-analysis of determinant factors influencing antibody response from vaccination of Ebola vaccine in non-human primate and human [7], which is a relevant paper to read to get a deeper insight and identify gaps for better formulation of our research question or purpose. We can still conduct systematic review and meta-analysis of Ebola vaccine because we evaluate safety as a different outcome and different population (only human).

#### Inclusion and exclusion criteria

Eligibility criteria are based on the PICO approach, study design, and date. Exclusion criteria mostly are unrelated, duplicated, unavailable full texts, or abstract-only papers. These exclusions should be stated in advance to refrain the researcher from bias. The inclusion criteria would be articles with the target patients, investigated interventions, or the comparison between two studied interventions. Briefly, it would be articles which contain information answering our research question. But the most important is that it should be clear and sufficient information, including positive or negative, to answer the question.

**Fig. 2:**
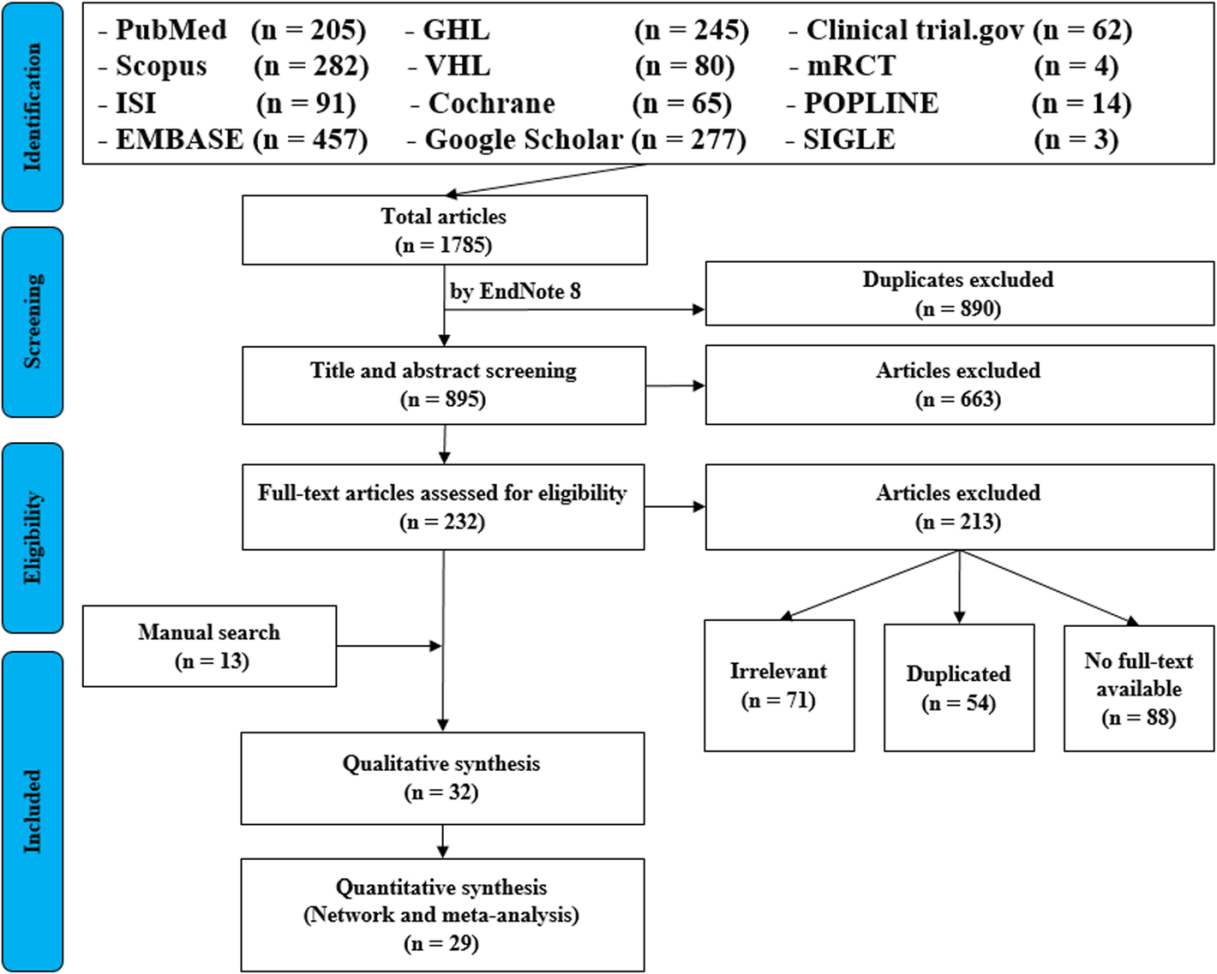
PRISMA flow diagram of studies’ screening and selection

For the topic we have chosen, we can make inclusion criteria: (1) any clinical trial evaluating the safety of Ebola vaccine and (2) no restriction regarding country, patient age, race, gender, publication language, and date. Exclusion criteria are as follows: (1) study of Ebola vaccine in non-human subjects or in vitro studies; (2) study with data not reliably extracted, duplicate, or overlapping data; (3) abstract-only papers as preceding papers, conference, editorial, and author response theses and books; (4) articles without available full text available; and (5) case reports, case series, and systematic review studies. The PRISMA flow diagram template that is used in SR/MA studies can be found in Fig. 2.

#### Search strategy

A standard search strategy is used in PubMed, then later it is modified according to each specific database to get the best relevant results. The basic search strategy is built based on the research question formulation (i.e., PICO or PICOS). Search strategies are constructed to include free-text terms (e.g., in the title and abstract) and any appropriate subject indexing (e.g., MeSH) expected to retrieve eligible studies, with the help of an expert in the review topic field or an information specialist. Additionally, we advise not to use terms for the Outcomes as their inclusion might hinder the database being searched to retrieve eligible studies because the used outcome is not mentioned obviously in the articles.

The improvement of the search term is made while doing a trial search and looking for another relevant term within each concept from retrieved papers. To search for a clinical trial, we can use these descriptors in PubMed: “clinical trial”[Publication Type] OR “clinical trials as topic”[MeSH terms] OR “clinical trial”[All Fields]. After some rounds of trial and refinement of search term, we formulate the final search term for PubMed as follows: (ebola OR ebola virus OR ebola virus disease OR EVD) AND (vaccine OR vaccination OR vaccinated OR immunization) AND (“clinical trial”[Publication Type] OR “clinical trials as topic”[MeSH Terms] OR “clinical trial”[All Fields]). Because the study for this topic is limited, we do not include outcome term (safety and immunogenicity) in the search term to capture more studies.

#### Search databases, import all results to a library, and exporting to an excel sheet

According to the AMSTAR guidelines, at least two databases have to be searched in the SR/MA [8], but as you increase the number of searched databases, you get much yield and more accurate and comprehensive results. The ordering of the databases depends mostly on the review questions; being in a study of clinical trials, you will rely mostly on Cochrane, mRCTs, or International Clinical Trials Registry Platform (ICTRP). Here, we propose 12 databases (PubMed, Scopus, Web of Science, EMBASE, GHL, VHL, Cochrane, Google Scholar, Clinical trials.gov, mRCTs, POPLINE, and SIGLE), which help to cover almost all published articles in tropical medicine and other health-related fields. Among those databases, POPLINE focuses on reproductive health. Researchers should consider to choose relevant database according to the research topic. Some databases do not support the use of Boolean or quotation; otherwise, there are some databases that have special searching way. Therefore, we need to modify the initial search terms for each database to get appreciated results; therefore, manipulation guides for each online database searches are presented in Additional file 5: Table S2. The detailed search strategy for each database is found in Additional file 5: Table S3. The search term that we created in PubMed needs customization based on a specific characteristic of the database. An example for Google Scholar advanced search for our topic is as follows:

1. With all of the words: ebola virus
  With at least one of the words: vaccine vaccination vaccinated immunization
  Where my words occur: in the title of the article
2. With all of the words: EVD
  With at least one of the words: vaccine vaccination vaccinated immunization
  Where my words occur: in the title of the article

Finally, all records are collected into one Endnote library in order to delete duplicates and then to it export into an excel sheet. Using remove duplicating function with two options is mandatory. All references which have (1) the same title and author, and published in the same year, and (2) the same title and author, and published in the same journal, would be deleted. References remaining after this step should be exported to an excel file with essential information for screening. These could be the authors’ names, publication year, journal, DOI, URL link, and abstract.

#### Protocol writing and registration

Protocol registration at an early stage guarantees transparency in the research process and protects from duplication problems. Besides, it is considered a documented proof of team plan of action, research question, eligibility criteria, intervention/exposure, quality assessment, and pre-analysis plan. It is recommended that researchers send it to the principal investigator (PI) to revise it, then upload it to registry sites. There are many registry sites available for SR/MA like those proposed by Cochrane and Campbell collaborations; however, we recommend registering the protocol into PROSPERO as it is easier. The layout of a protocol template, according to PROSPERO, can be found in Additional file 5: File S1.

#### Title and abstract screening

Decisions to select retrieved articles for further assessment are based on eligibility criteria, to minimize the chance of including non-relevant articles. According to the Cochrane guidance, two reviewers are a must to do this step, but as for beginners and junior researchers, this might be tiresome; thus, we propose based on our experience that at least three reviewers should work independently to reduce the chance of error, particularly in teams with a large number of authors to add more scrutiny and ensure proper conduct. Mostly, the quality with three reviewers would be better than two, as two only would have different opinions from each other, so they cannot decide, while the third opinion is crucial. And here are some examples of systematic reviews which we conducted following the same strategy (by a different group of researchers in our research group) and published successfully, and they feature relevant ideas to tropical medicine and disease [9–11].

In this step, duplications will be removed manually whenever the reviewers find them out. When there is a doubt about an article decision, the team should be inclusive rather than exclusive, until the main leader or PI makes a decision after discussion and consensus. All excluded records should be given exclusion reasons.

#### Full text downloading and screening

Many search engines provide links for free to access full-text articles. In case not found, we can search in some research websites as ResearchGate, which offer an option of direct full-text request from authors. Additionally, exploring archives of wanted journals, or contacting PI to purchase it if available. Similarly, 2–3 reviewers work independently to decide about included full texts according to eligibility criteria, with reporting exclusion reasons of articles. In case any disagreement has occurred, the final decision has to be made by discussion.

#### Manual search

One has to exhaust all possibilities to reduce bias by performing an explicit hand-searching for retrieval of reports that may have been dropped from first search [12]. We apply five methods to make manual searching: searching references from included studies/reviews, contacting authors and experts, and looking at related articles/cited articles in PubMed and Google Scholar.

We describe here three consecutive methods to increase and refine the yield of manual searching: firstly, searching reference lists of included articles; secondly, performing what is known as citation tracking in which the reviewers track all the articles that cite each one of the included articles, and this might involve electronic searching of databases; and thirdly, similar to the citation tracking, we follow all “related to” or “similar” articles. Each of the abovementioned methods can be performed by 2–3 independent reviewers, and all the possible relevant article must undergo further scrutiny against the inclusion criteria, after following the same records yielded from electronic databases, i.e., title/abstract and full-text screening.

We propose an independent reviewing by assigning each member of the teams a “tag” and a distinct method, to compile all the results at the end for comparison of differences and discussion and to maximize the retrieval and minimize the bias. Similarly, the number of included articles has to be stated before addition to the overall included records.

#### Data extraction and quality assessment

This step entitles data collection from included full-texts in a structured extraction excel sheet, which is previously pilot-tested for extraction using some random studies. We recommend extracting both adjusted and non-adjusted data because it gives the most allowed confounding factor to be used in the analysis by pooling them later [13]. The process of extraction should be executed by 2–3 independent reviewers. Mostly, the sheet is classified into the study and patient characteristics, outcomes, and quality assessment (QA) tool.

Data presented in graphs should be extracted by software tools such as Web plot digitizer [14]. Most of the equations that can be used in extraction prior to analysis and estimation of standard deviation (SD) from other variables is found inside Additional file 5: File S2 with their references as Hozo et al. [15], Xiang et al. [16], and Rijkom et al. [17]. A variety of tools are available for the QA, depending on the design: ROB-2 Cochrane tool for randomized controlled trials [18] which is presented as Additional file 1: Figure S1 and Additional file 2: Figure S2—from a previous published article data—[19], NIH tool for observational and cross-sectional studies [20], ROBINS-I tool for non-randomize trials [21], QUADAS-2 tool for diagnostic studies, QUIPS tool for prognostic studies, CARE tool for case reports, and ToxRtool for in vivo and in vitro studies. We recommend that 2–3 reviewers independently assess the quality of the studies and add to the data extraction form before the inclusion into the analysis to reduce the risk of bias. In the NIH tool for observational studies—cohort and cross-sectional—as in this EBOLA case, to evaluate the risk of bias, reviewers should rate each of the 14 items into dichotomous variables: yes, no, or not applicable. An overall score is calculated by adding all the items scores as yes equals one, while no and NA equals zero. A score will be given for every paper to classify them as poor, fair, or good conducted studies, where a score from 0–5 was considered poor, 6–9 as fair, and 10–14 as good.

In the EBOLA case example above, authors can extract the following information: name of authors, country of patients, year of publication, study design (case report, cohort study, or clinical trial or RCT), sample size, the infected point of time after EBOLA infection, follow-up interval after vaccination time, efficacy, safety, adverse effects after vaccinations, and QA sheet (Additional file 6: Data S1).

#### Data checking

Due to the expected human error and bias, we recommend a data checking step, in which every included article is compared with its counterpart in an extraction sheet by evidence photos, to detect mistakes in data. We advise assigning articles to 2–3 independent reviewers, ideally not the ones who performed the extraction of those articles. When resources are limited, each reviewer is assigned a different article than the one he extracted in the previous stage.

#### Statistical analysis

Investigators use different methods for combining and summarizing findings of included studies. Before analysis, there is an important step called cleaning of data in the extraction sheet, where the analyst organizes extraction sheet data in a form that can be read by analytical software. The analysis consists of 2 types namely qualitative and quantitative analysis. Qualitative analysis mostly describes data in SR studies, while quantitative analysis consists of two main types: MA and network meta-analysis (NMA). Subgroup, sensitivity, cumulative analyses, and meta-regression are appropriate for testing whether the results are consistent or not and investigating the effect of certain confounders on the outcome and finding the best predictors. Publication bias should be assessed to investigate the presence of missing studies which can affect the summary.

To illustrate basic meta-analysis, we provide an imaginary data for the research question about Ebola vaccine safety (in terms of adverse events, 14 days after injection) and immunogenicity (Ebola virus antibodies rise in geometric mean titer, 6 months after injection). Assuming that from searching and data extraction, we decided to do an analysis to evaluate Ebola vaccine “A” safety and immunogenicity. Other Ebola vaccines were not meta-analyzed because of the limited number of studies (instead, it will be included for narrative review). The imaginary data for vaccine safety meta-analysis can be accessed in Additional file 7: Data S2. To do the meta-analysis, we can use free software, such as RevMan [22] or R package meta [23]. In this example, we will use the R package meta. The tutorial of meta package can be accessed through “General Package for Meta-Analysis” tutorial pdf [23]. The R codes and its guidance for meta-analysis done can be found in Additional file 5: File S3.

For the analysis, we assume that the study is heterogenous in nature; therefore, we choose a random effect model. We did an analysis on the safety of Ebola vaccine A. From the data table, we can see some adverse events occurring after intramuscular injection of vaccine A to the subject of the study. Suppose that we include six studies that fulfill our inclusion criteria. We can do a meta-analysis for each of the adverse events extracted from the studies, for example, arthralgia, from the results of random effect meta-analysis using the R meta package.

From the results shown in Additional file 3: Figure S3, we can see that the odds ratio (OR) of arthralgia is 1.06 (0.79; 1.42), *p* value = 0.71, which means that there is no association between the intramuscular injection of Ebola vaccine A and arthralgia, as the OR is almost one, and besides, the *P* value is insignificant as it is > 0.05.

**Fig. 3:**
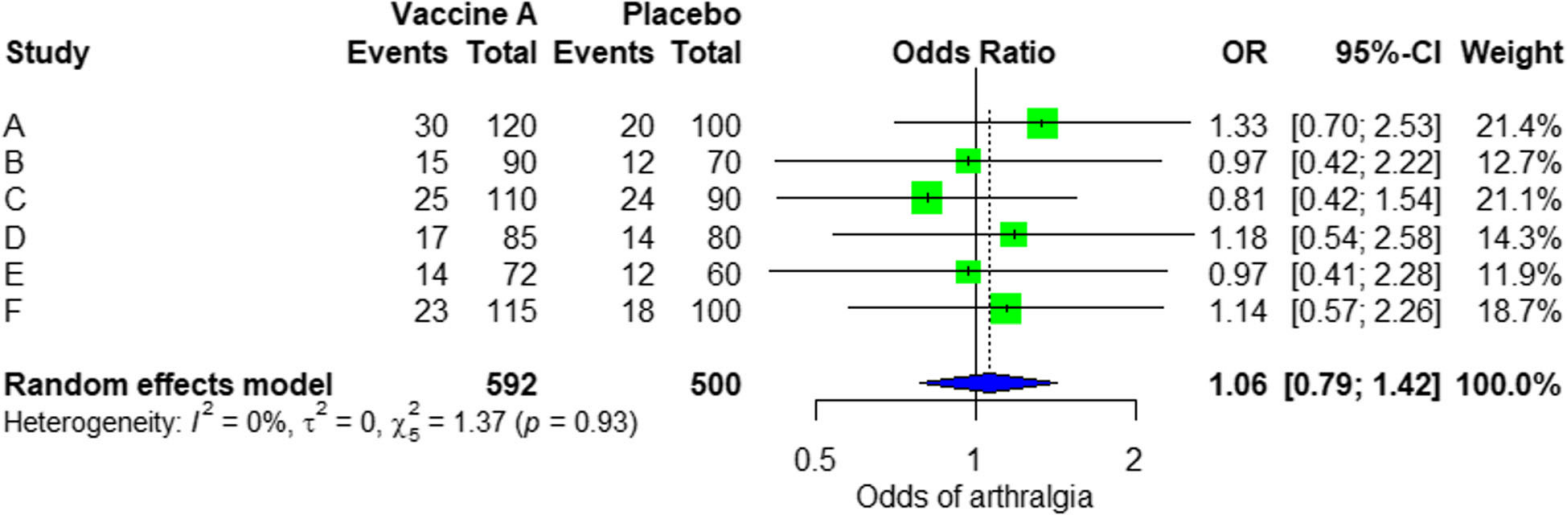
Random effect model forest plot for comparison of vaccine A versus placebo

In the meta-analysis, we can also visualize the results in a forest plot. It is shown in Fig. 3 an example of a forest plot from the simulated analysis.

From the forest plot, we can see six studies (A to F) and their respective OR (95% CI). The green box represents the effect size (in this case, OR) of each study. The bigger the box means the study weighted more (i.e., bigger sample size). The blue diamond shape represents the pooled OR of the six studies. We can see the blue diamond cross the vertical line OR = 1, which indicates no significance for the association as the diamond almost equalized in both sides. We can confirm this also from the 95% confidence interval that includes one and the *p* value > 0.05.

**Fig. 4:**
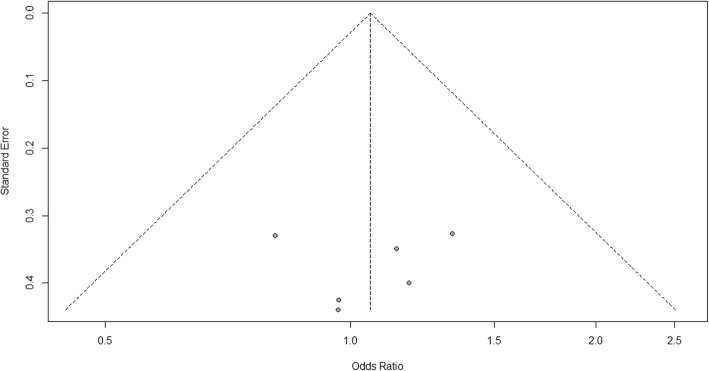
Publication bias funnel plot for comparison of vaccine A versus placebo

For heterogeneity, we see that *I*^2^ = 0%, which means no heterogeneity is detected; the study is relatively homogenous (it is rare in the real study). To evaluate publication bias related to the meta-analysis of adverse events of arthralgia, we can use the metabias function from the R meta package (Additional file 4: Figure S4) and visualization using a funnel plot. The results of publication bias are demonstrated in Fig. 4. We see that the *p* value associated with this test is 0.74, indicating symmetry of the funnel plot. We can confirm it by looking at the funnel plot.

Looking at the funnel plot, the number of studies at the left and right side of the funnel plot is the same; therefore, the plot is symmetry, indicating no publication bias detected.

Sensitivity analysis is a procedure used to discover how different values of an independent variable will influence the significance of a particular dependent variable by removing one study from MA. If all included study *p* values are < 0.05, hence, removing any study will not change the significant association. It is only performed when there is a significant association, so if the *p* value of MA done is 0.7—more than one—the sensitivity analysis is not needed for this case study example. If there are 2 studies with *p* value > 0.05, removing any of the two studies will result in a loss of the significance.

#### Double data checking

For more assurance on the quality of results, the analyzed data should be rechecked from full-text data by evidence photos, to allow an obvious check for the PI of the study.

#### Manuscript writing, revision, and submission to a journal

Writing based on four scientific sections: introduction, methods, results, and discussion, mostly with a conclusion. Performing a characteristic table for study and patient characteristics is a mandatory step which can be found as a template in Additional file 5: Table S3.

After finishing the manuscript writing, characteristics table, and PRISMA flow diagram, the team should send it to the PI to revise it well and reply to his comments and, finally, choose a suitable journal for the manuscript which fits with considerable impact factor and fitting field. We need to pay attention by reading the author guidelines of journals before submitting the manuscript.

## Discussion

The role of evidence-based medicine in biomedical research is rapidly growing. SR/MAs are also increasing in the medical literature. This paper has sought to provide a comprehensive approach to enable reviewers to produce high-quality SR/MAs. We hope that readers could gain general knowledge about how to conduct a SR/MA and have the confidence to perform one, although this kind of study requires complex steps compared to narrative reviews.

Having the basic steps for conduction of MA, there are many advanced steps that are applied for certain specific purposes. One of these steps is meta-regression which is performed to investigate the association of any confounder and the results of the MA. Furthermore, there are other types rather than the standard MA like NMA and MA. In NMA, we investigate the difference between several comparisons when there were not enough data to enable standard meta-analysis. It uses both direct and indirect comparisons to conclude what is the best between the competitors. On the other hand, mega MA or MA of patients tend to summarize the results of independent studies by using its individual subject data. As a more detailed analysis can be done, it is useful in conducting repeated measure analysis and time-to-event analysis. Moreover, it can perform analysis of variance and multiple regression analysis; however, it requires homogenous dataset and it is time-consuming in conduct [24].

## Conclusions

Systematic review/meta-analysis steps include development of research question and its validation, forming criteria, search strategy, searching databases, importing all results to a library and exporting to an excel sheet, protocol writing and registration, title and abstract screening, full-text screening, manual searching, extracting data and assessing its quality, data checking, conducting statistical analysis, double data checking, manuscript writing, revising, and submitting to a journal.

## Additional files

Additional file 1: **Figure S1.** Risk of bias assessment graph of included randomized controlled trials. (TIF 20 kb)

Additional file 2: **Figure S2.** Risk of bias assessment summary. (TIF 69 kb)

Additional file 3: **Figure S3.** Arthralgia results of random effect meta-analysis using R meta package. (TIF 20 kb)

Additional file 4: **Figure S4.** Arthralgia linear regression test of funnel plot asymmetry using R meta package. (TIF 13 kb)

Additional file 5: **Table S1.** PRISMA 2009 Checklist. **Table S2.** Manipulation guides for online database searches. **Table S3.** Detailed search strategy for twelve database searches. **Table S4.** Baseline characteristics of the patients in the included studies. File S1. PROSPERO protocol template file. File S2. Extraction equations that can be used prior to analysis to get missed variables. File S3. R codes and its guidance for meta-analysis done for comparison between EBOLA vaccine A and placebo. (DOCX 49 kb)

Additional file 6: Data S1. Extraction and quality assessment data sheets for EBOLA case example. (XLSX 1368 kb)

Additional file 7: Data S2. Imaginary data for EBOLA case example. (XLSX 10 kb)

## Data Availability

Not applicable.

## Abbreviations

NMA: Network meta-analysis
PI: Principal investigator
PICO: Population, Intervention, Comparison, Outcome
PRISMA: Preferred Reporting Items for Systematic Review and Meta-analysis statement
QA: Quality assessment
SPIDER: Sample, Phenomenon of Interest, Design, Evaluation, Research type
SR/MAs: Systematic review and meta-analyses

## References

[1] Bello A, Wiebe N, Garg A, Tonelli M (2015). Evidence-based decision-making 2: systematic reviews and meta-analysis. Methods Mol Biol (Clifton, NJ)..

[2] Khan KS, Kunz R, Kleijnen J, Antes G (2003). Five steps to conducting a systematic review. J R Soc Med..

[3] Rys P, Wladysiuk M, Skrzekowska-Baran I, Malecki MT (2009). Review articles, systematic reviews and meta-analyses: which can be trusted?. Polskie Archiwum Medycyny Wewnetrznej..

[4] Higgins JPT, Green S. Cochrane Handbook for Systematic Reviews of Interventions Version 5.1.0 [updated March 2011]. 2011.

[5] Moher D, Liberati A, Tetzlaff J, Altman DG (2009). Preferred reporting items for systematic reviews and meta-analyses: the PRISMA statement. BMJ..

[6] Methley AM, Campbell S, Chew-Graham C, McNally R, Cheraghi-Sohi S (2014). PICO, PICOS and SPIDER: a comparison study of specificity and sensitivity in three search tools for qualitative systematic reviews. BMC Health Serv Res..

[7] Gross L, Lhomme E, Pasin C, Richert L, Thiebaut R (2018). Ebola vaccine development: systematic review of pre-clinical and clinical studies, and meta-analysis of determinants of antibody response variability after vaccination. Int J Infect Dis.

[8] Shea BJ, Reeves BC, Wells G, Thuku M, Hamel C, Moran J, ... Henry DA. AMSTAR 2: a critical appraisal tool for systematic reviews that include randomised or non-randomised studies of healthcare interventions, or both. BMJ. 2017;358:j4008. 10.1136/bmj.j4008 PMC5833365 28935701

[9] Giang HTN, Banno K, Minh LHN, Trinh LT, Loc LT, Eltobgy A (2018). Dengue hemophagocytic syndrome: a systematic review and meta-analysis on epidemiology, clinical signs, outcomes, and risk factors. Rev Med Virol..

[10] Morra ME, Altibi AMA, Iqtadar S, Minh LHN, Elawady SS, Hallab A (2018). Definitions for warning signs and signs of severe dengue according to the WHO 2009 classification: systematic review of literature. Rev Med Virol..

[11] Morra ME, Van Thanh L, Kamel MG, Ghazy AA, Altibi AMA, Dat LM (2018). Clinical outcomes of current medical approaches for Middle East respiratory syndrome: a systematic review and meta-analysis. Rev Med Virol..

[12] Vassar M, Atakpo P, Kash MJ (2016). Manual search approaches used by systematic reviewers in dermatology. Journal of the Medical Library Association: JMLA..

[13] Naunheim MR, Remenschneider AK, Scangas GA, Bunting GW, Deschler DG (2016). The effect of initial tracheoesophageal voice prosthesis size on postoperative complications and voice outcomes. Ann Otol Rhinol Laryngol..

[14] Rohatgi AJaiWa. Web Plot Digitizer. ht tp. 2014;2.

[15] Hozo SP, Djulbegovic B, Hozo I (2005). Estimating the mean and variance from the median, range, and the size of a sample. BMC Med Res Methodol.

[16] Wan X, Wang W, Liu J, Tong T (2014). Estimating the sample mean and standard deviation from the sample size, median, range and/or interquartile range. BMC Med Res Methodol.

[17] Van Rijkom HM, Truin GJ, Van’t Hof MA. A meta-analysis of clinical studies on the caries-inhibiting effect of fluoride gel treatment. Carries Res. 1998;32(2):83–92. 10.1159/000016436 9544855

[18] Higgins JP, Altman DG, Gotzsche PC, Juni P, Moher D, Oxman AD (2011). The Cochrane Collaboration's tool for assessing risk of bias in randomised trials. BMJ..

[19] Tawfik GM, Tieu TM, Ghozy S, Makram OM, Samuel P, Abdelaal A (2018). Speech efficacy, safety and factors affecting lifetime of voice prostheses in patients with laryngeal cancer: a systematic review and network meta-analysis of randomized controlled trials. J Clin Oncol.

[20] Wannemuehler TJ, Lobo BC, Johnson JD, Deig CR, Ting JY, Gregory RL (2016). Vibratory stimulus reduces in vitro biofilm formation on tracheoesophageal voice prostheses. Laryngoscope..

[21] Sterne JAC, Hernán MA, Reeves BC, Savović J, Berkman ND, Viswanathan M, et al. ROBINS-I: a tool for assessing risk of bias in non-randomised studies of interventions. BMJ. 2016;355. 10.1136/bmj.i4919 PMC5062054 27733354

[22] RevMan The Cochrane Collaboration %J Copenhagen TNCCTCC. Review Manager (RevMan). 5.0. 2008.

[23] Schwarzer GJRn (2007). meta: An R package for meta-analysis.

[24] Simms LLH. Meta-analysis versus mega-analysis: is there a difference? Oral budesonide for the maintenance of remission in Crohn’s disease: Faculty of Graduate Studies, University of Western Ontario; 1998.

