# Supplementary figures and images for: A step by step guide for conducting a systematic review and meta-analysis with simulation data
Source: Trop Med Health. 2019 Aug 1;47:46. doi: 10.1186/s41182-019-0165-6 (PMC6670166; doi:10.1186/s41182-019-0165-6)

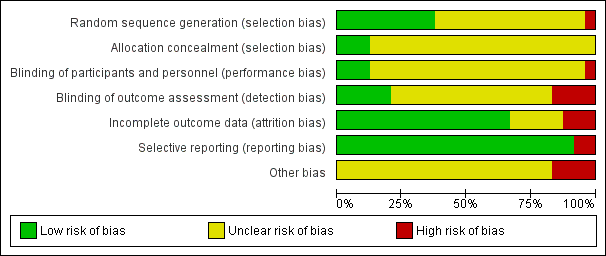

Supplement: Supplementary file 1 — Figure S1. Risk of bias assessment graph of included randomized controlled trials. (TIF 20 kb) [file 41182_2019_165_MOESM1_ESM.tif]

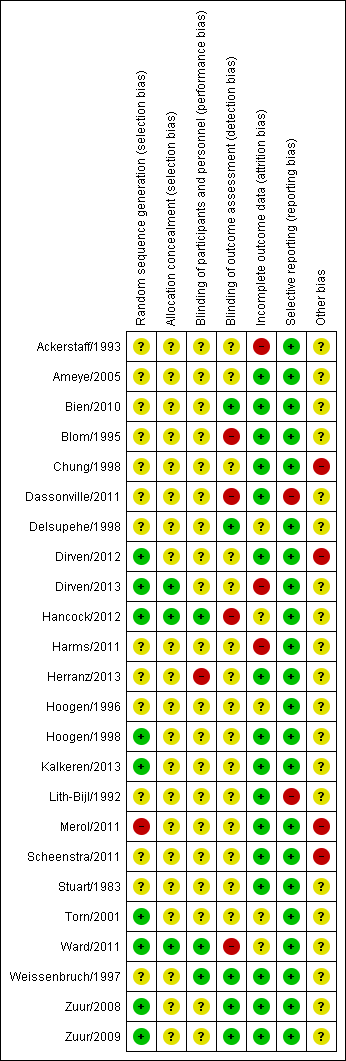

Supplement: Supplementary file 2 — Figure S2. Risk of bias assessment summary. (TIF 69 kb) [file 41182_2019_165_MOESM2_ESM.tif]

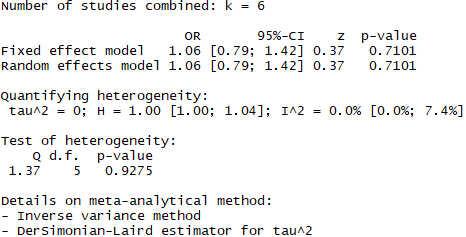

Supplement: Supplementary file 3 — Figure S3. Arthralgia results of random effect meta-analysis using R meta package. (TIF 20 kb) [file 41182_2019_165_MOESM3_ESM.tif]

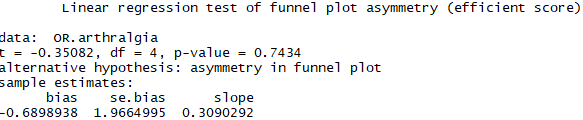

Supplement: Supplementary file 4 — Figure S4. Arthralgia linear regression test of funnel plot asymmetry using R meta package. (TIF 13 kb) [file 41182_2019_165_MOESM4_ESM.tif]
