# Supplementary material for: A step by step guide for conducting a systematic review and meta-analysis with simulation data
Source: Trop Med Health. 2019 Aug 1;47:46. doi: 10.1186/s41182-019-0165-6 (PMC6670166; doi:10.1186/s41182-019-0165-6)
Supplement: Supplementary file 5 — Table S1. PRISMA 2009 Checklist. Table S2. Manipulation guides for online database searches. Table S3. Detailed search strategy for twelve database searches. Table S4. Baseline characteristics of the patients in the included studies. File S1. PROSPERO protocol template file. File S2. Extraction equations that can be used prior to analysis to get missed variables. File S3. R codes and its guidance for meta-analysis done for comparison between EBOLA vaccine A and placebo. (DOCX 49 kb) [file 41182_2019_165_MOESM5_ESM.docx]

**Supplementary Figure legends**

Supplementary Figure 1. Risk of bias assessment graph of included randomized controlled trials.

Supplementary Figure 2. Risk of bias assessment summary.

Supplementary Figure 3. Arthralgia results of random effect meta-analysis using R meta package.

Supplementary Figure 4. Arthralgia linear regression test of funnel plot asymmetry using R meta package.

**Supplementary Table legends**

Supplementary Table 1. PRISMA 2009 Checklist.

Supplementary Table 2. Manipulation guides for online database searches.

Supplementary Table 3. Detailed search strategy for tweleve database searches.

Supplementary Table 4. Baseline characteristics of the patients in the included studies.

**Supplementary File legends**

Supplementary File 1. Prospero protocol template file.

Supplementary File 2. Extraction equations that can be used prior to analysis to get missed variables.

Supplementary File 3. R codes and its guidance for meta-analysis done for comparison between EBOLA vaccine A and placebo.

Supplementary Data 1. Extraction and quality assessment sheets for EBOLA case example.

Supplementary Data 2. Imaginary data for EBOLA case example.

***Supplementary* *Table 1. PRISMA 2009 Checklist***

| ***Section/topic*** | ***#*** | ***Checklist item*** | ***Reported on page #*** |
| --- | --- | --- | --- |
| **TITLE** | | |  |
| **Title** | 1 | Identify the report as a systematic review, meta-analysis, or both. |  |
| **ABSTRACT** | | |  |
| **Structured summary** | 2 | Provide a structured summary including, as applicable: background; objectives; data sources; study eligibility criteria, participants, and interventions; study appraisal and synthesis methods; results; limitations; conclusions and implications of key findings; systematic review registration number. |  |
| **INTRODUCTION** | | |  |
| **Rationale** | 3 | Describe the rationale for the review in the context of what is already known. |  |
| **Objectives** | 4 | Provide an explicit statement of questions being addressed with reference to participants, interventions, comparisons, outcomes, and study design (PICOS). |  |
| **METHODS** | | |  |
| **Protocol and registration** | 5 | Indicate if a review protocol exists, if and where it can be accessed (e.g., Web address), and, if available, provide registration information including registration number. |  |
| **Eligibility criteria** | 6 | Specify study characteristics (e.g., PICOS, length of follow-up) and report characteristics (e.g., years considered, language, publication status) used as criteria for eligibility, giving rationale. |  |
| **Information sources** | 7 | Describe all information sources (e.g., databases with dates of coverage, contact with study authors to identify additional studies) in the search and date last searched. |  |
| **Search** | 8 | Present full electronic search strategy for at least one database, including any limits used, such that it could be repeated. |  |
| **Study selection** | 9 | State the process for selecting studies (i.e., screening, eligibility, included in systematic review, and, if applicable, included in the meta-analysis). |  |
| **Data collection process** | 10 | Describe method of data extraction from reports (e.g., piloted forms, independently, in duplicate) and any processes for obtaining and confirming data from investigators. |  |
| **Data items** | 11 | List and define all variables for which data were sought (e.g., PICOS, funding sources) and any assumptions and simplifications made. |  |
| **Risk of bias in individual studies** | 12 | Describe methods used for assessing risk of bias of individual studies (including specification of whether this was done at the study or outcome level), and how this information is to be used in any data synthesis. |  |
| **Summary measures** | 13 | State the principal summary measures (e.g., risk ratio, difference in means). |  |
| **Synthesis of results** | 14 | Describe the methods of handling data and combining results of studies, if done, including measures of consistency (e.g., I^2^) for each meta-analysis. |  |
| **Risk of bias across studies** | 15 | Specify any assessment of risk of bias that may affect the cumulative evidence (e.g., publication bias, selective reporting within studies). |  |
| **Additional analyses** | 16 | Describe methods of additional analyses (e.g., sensitivity or subgroup analyses, meta-regression), if done, indicating which were pre-specified. |  |
| **RESULTS** | | |  |
| **Study selection** | 17 | Give numbers of studies screened, assessed for eligibility, and included in the review, with reasons for exclusions at each stage, ideally with a flow diagram. |  |
| **Study characteristics** | 18 | For each study, present characteristics for which data were extracted (e.g., study size, PICOS, follow-up period) and provide the citations. |  |
| **Risk of bias within studies** | 19 | Present data on risk of bias of each study and, if available, any outcome level assessment (see item 12). |  |
| **Results of individual studies** | 20 | For all outcomes considered (benefits or harms), present, for each study: (a) simple summary data for each intervention group (b) effect estimates and confidence intervals, ideally with a forest plot. |  |
| **Synthesis of results** | 21 | Present results of each meta-analysis done, including confidence intervals and measures of consistency. |  |
| **Risk of bias across studies** | 22 | Present results of any assessment of risk of bias across studies (see Item 15). |  |
| **Additional analysis** | 23 | Give results of additional analyses, if done (e.g., sensitivity or subgroup analyses, meta-regression [see Item 16]). |  |
| **DISCUSSION** | | |  |
| **Summary of evidence** | 24 | Summarize the main findings including the strength of evidence for each main outcome; consider their relevance to key groups (e.g., healthcare providers, users, and policy makers). |  |
| **Limitations** | 25 | Discuss limitations at study and outcome level (e.g., risk of bias), and at review-level (e.g., incomplete retrieval of identified research, reporting bias). |  |
| **Conclusions** | 26 | Provide a general interpretation of the results in the context of other evidence, and implications for future research. |  |
| **FUNDING** | | |  |
| **Funding** | 27 | Describe sources of funding for the systematic review and other support (e.g., supply of data); role of funders for the systematic review. |  |

***Supplementary Table 2. Manipulation guides for online database searches***

| ***No.*** | ***Databases (Total 12)*** | ***Explanation*** |
| --- | --- | --- |
| **1** | **PubMed** | 1. Perform PubMed search.  2. Choose the reports that the reviewer wants to export (if reviewer does not choose any report, PubMed system will automatically select all).0  3. Click on Send To, choose File.  4. Choose MEDLINE format.  5. Click Create File.  6. Move PubMed file to a suitable directory. |
| **2** | **Scopus** | 1. Perform Scopus search.  2. Select all articles.  3. Click on Export.  4. On the new interface, choose RIS format.  5. At Choose the Information to export, choose All available information.  6. Click on Export.  7. Move Scopus file to a suitable directory. |
| **3** | **ISI (WOS)** | 1. Perform ISI search.  2. Select all articles.  3. In the Save to panel, click on EndNote.  4. Move ISI file to a suitable directory. |
| **4** | **GHL** | 1. Perform WHO Global Health Library search.  2. Click on Send Results, tab Export.  3. Choose All references.  4. Choose RIS for export format.  5. Click on Send.  6. Move WHO-GHL file to a suitable directory. |
| **5** | **VHL** | 1. Perform VHL search.  2. Click on Export button (next to the Print button).  3. At Export format, choose RIS.  4. At Export, choose All references.  5. Move VHL file to a suitable directory. |
| **6** | **POPLINE** | 1. At Export Search Results, click on RIS to download to get Popline file for  2. Move Popline file to suitable directory.  3. Perform the same task for the rest pages until reviewers get all necessary files.  4. Finally, move all files to a suitable directory. |
| **7** | **Cochrane** | 1. Perform Cochrane search. |
| **8** | **EMBASE** | 1. Perform EMBASE search. |
| **9** | **mRCT** | 1. Perform mRCT search. |
| **10** | **Clinical trial.gov** | 1. Perform Clinical trial search. |
| **11** | **Google Scholar** | 1. Perform Google Scholar search.  2. Click on Settings.  3. At Results per page, change to 20.  4. At Bibliography manager, choose Show links to import citations into EndNote, then click Save.  5. On Navigation Toolbar in Google chrome browser, click on the Zotero icon.  6. Choose Select All in the drop down window, and then click OK.  7. Move to the next pages and perform the same task (step (5) and (6)).  8. At the bottom-right corner of the browser (on Add-on Bar), click on the Zotero  9. On the new interface, click on Actions button, choose Export library.  10. Choose RIS format.  11. Click OK.  12. Move the exported Google Scholar file to a suitable directory. |
| **12** | **SIGLE** | 1. Perform SIGLE search.  2. Click on the Zotero icon in the URL bar to export references to Zotero.  3. Then, do the same steps described above for Google Scholar. |

***Supplementary Table 3. Detailed search strategy for twelve database searches***

| ***No.*** | ***Databases (Total 12)*** | ***Search Terms*** | ***Results***  ***Total = 1785*** |
| --- | --- | --- | --- |
| **1** | **PubMed** | (ebola OR ebola virus OR ebola virus disease OR EVD) AND (vaccine OR vaccination OR vaccinated OR immunization) AND ("clinical trial"[Publication Type] OR "clinical trials as topic"[MeSH Terms] OR "clinical trial"[All Fields]) | **205** |
| **2** | **Scopus** | TITLE-ABS-KEY ( ( ebola  OR  ebola  AND virus  OR  ebola  AND virus  AND disease  OR  evd )  AND  ( vaccine  OR  vaccination  OR  vaccinated  OR  immunization )  AND  "clinical trial" ) | **282** |
| **3** | **ISI (WOS)** | (ebola OR ebola virus OR ebola virus disease OR EVD) AND (vaccine OR vaccination OR vaccinated OR immunization) AND "clinical trial" | **91** |
| **4** | **EMBASE** |  | **457** |
| **5** | **GHL** |  | **245** |
| **6** | **VHL** |  | **80** |
| **7** | **Cochrane** |  | **65** |
| **8** | **Google Scholar** | Where my words occur: in the title of the article: 1. With all of the words: ebola virus With at least one of the words: vaccine vaccination vaccinated immunization 2. With all of the words: EVD With at least one of the words: vaccine vaccination vaccinated immunization | **272 + 5 = 277** |
| **9** | **Clinical trial.gov** | Condition or disease: ebola OR ebola virus OR ebola virus disease OR EVD  Other terms: vaccine OR vaccination OR vaccinated OR immunization | 62 |
| **10** | **mRCT** | (ebola OR ebola virus OR ebola virus disease OR EVD) AND (vaccine OR vaccination OR vaccinated OR immunization) AND "clinical trial" | 4 |
| **11** | **POPLINE** |  | **14** |
| **12** | **SIGLE** | (ebola OR EVD) AND (vaccine) | **3** |

***Supplementary Table 4. Study and patient characteristics of the included studies***

| ***Author/Year/ Country of patients*** | ***Study design*** | ***Sample size*** | ***Mean age (years)*** | ***Male event (%)*** | ***Study Arms (event/total) (Arthralgia outcome)*** | | ***Quality assessment grade (Score)*** | ***Follow up (months)*** |
| --- | --- | --- | --- | --- | --- | --- | --- | --- |
|  |  |  |  |  | ***Vaccine*** | ***Placebo*** |  |  |
| Study1/2016/Japan | Cohort | 220 | 32 | 100 (45.5) | 30/120 | 20/100 | Good (10) | 3 |
| Study2/2018/Vietnam | Cohort | 160 | 35 | 95 (59.4) | 15/90 | 12/70 | Fair (7) | 5 |
| Study3/2017/USA | Cohort | 200 | 30 | 100 (50) | 25/110 | 24/90 | Good (11) | 8 |
| Study4/2015/Egypt | Cross-sectional | 165 | 25 | 50 (30.3) | 17/85 | 14/80 | Good (12) | 2 |
| Study5/2012/India | Cross-sectional | 132 | 50 | 70 (53) | 14/72 | 12/60 | Fair (9) | 7 |
| Study6/2012/UK | Cross-sectional | 225 | 53 | 150 (66.7) | 23/115 | 18/100 | Poor (5) | 7 |

| ***Supplementary File 1. Prospero protocol template file***  **PROSPERO International prospective register of systematic reviews** |
| --- |

| Review title and timescale | |
| --- | --- |
| 1 | Review titleGive the working title of the review. This must be in English. Ideally it should state succinctly the interventions or exposures being reviewed and the associated health or social problem being addressed in the review. |
| 2 | Original language titleFor reviews in languages other than English, this field should be used to enter the title in the language of the review. This will be displayed together with the English language title. |
| 3 | Anticipated or actual start dateGive the date when the systematic review commenced, or is expected to commence. |
| 4 | Anticipated completion dateGive the date by which the review is expected to be completed. |
| 5 | Stage of review at time of this submissionIndicate the stage of progress of the review by ticking the relevant boxes. Reviews that have progressed beyond the point of completing data extraction at the time of initial registration are not eligible for inclusion in PROSPERO. This field should be updated when any amendments are made to a published record. |
|  | \| The review has not yet started \| **×** \|  \|  \| \| --- \| --- \| --- \| --- \| \|  \| \|  \|  \| \| Review stage \| \| Started \| Completed \| \| Preliminary searches \| \| Yes \| Yes \| \| Piloting of the study selection process \| \| Yes \| Yes \| \| Formal screening of search results against eligibility criteria \| \| Yes \| No \| \| Data extraction \| \| No \| No \| \| Risk of bias (quality) assessment \| \| No \| No \| \| Data analysis \| \| No \| No \| |
|  | Provide any other relevant information about the stage of the review here. |
| Review team details | |
| 6 | Named contactThe named contact acts as the guarantor for the accuracy of the information presented in the register record. |
| 7 | Named contact emailEnter the electronic mail address of the named contact. |
| 8 | Named contact addressEnter the full postal address for the named contact. |
| 9 | Named contact phone numberEnter the telephone number for the named contact, including international dialing code. |
| 10 | Organisational affiliation of the reviewFull title of the organisational affiliations for this review, and website address if available. This field may be completed as 'None' if the review is not affiliated to any organisation.Website address: |
| 11 | Review team members and their organisational affiliationsGive the title, first name and last name of all members of the team working directly on the review. Give the organisational affiliations of each member of the review team. |
|  | \| Title \| First name \| Last name \| Affiliation \| \| --- \| --- \| --- \| --- \| \|  \|  \|  \|  \| |
| 12 | Funding sources/sponsorsGive details of the individuals, organizations, groups or other legal entities who take responsibility for initiating, managing, sponsoring and/or financing the review. Any unique identification numbers assigned to the review by the individuals or bodies listed should be included. |
| 13 | Conflicts of interestList any conditions that could lead to actual or perceived undue influence on judgements concerning the main topic investigated in the review.Are there any actual or potential conflicts of interest? |
| 14 | CollaboratorsGive the name, affiliation and role of any individuals or organisations who are working on the review but who are not listed as review team members. |
|  | \| Title \| First name \| Last name \| Organisation details \| \| --- \| --- \| --- \| --- \| |
| Review methods | |
| 15 | Review question(s)State the question(s) to be addressed / review objectives. Please complete a separate box for each question. |
| 16 | SearchesGive details of the sources to be searched, and any restrictions (e.g. language or publication period). The full search strategy is not required, but may be supplied as a link or attachment. |
| 17 | URL to search strategyIf you have one, give the link to your search strategy here. Alternatively you can e-mail this to PROSPERO and we will store and link to it. |
| 18 | Condition or domain being studiedGive a short description of the disease, condition or healthcare domain being studied. This could include health and wellbeing outcomes. |
| 19 | Participants/populationGive summary criteria for the participants or populations being studied by the review. The preferred format includes details of both inclusion and exclusion criteria. |
| 20 | Intervention(s), exposure(s)Give full and clear descriptions of the nature of the interventions or the exposures to be reviewed |
| 21 | Comparator(s)/controlWhere relevant, give details of the alternatives against which the main subject/topic of the review will be compared (e.g. another intervention or a non-exposed control group). |
| 22 | Types of study to be includedGive details of the study designs to be included in the review. If there are no restrictions on the types of study design eligible for inclusion, this should be stated. |
| 23 | ContextGive summary details of the setting and other relevant characteristics which help define the inclusion or exclusion criteria. |
| 24 | Primary outcome(s)Give the most important outcomes.Give information on timing and effect measures, as appropriate. |
| 25 | Secondary outcomesList any additional outcomes that will be addressed. If there are no secondary outcomes enter None. |
|  | Give information on timing and effect measures, as appropriate. |
| 26 | Data extraction (selection and coding)Give the procedure for selecting studies for the review and extracting data, including the number of researchers involved and how discrepancies will be resolved. List the data to be extracted. |
| 27 | Risk of bias (quality) assessmentState whether and how risk of bias will be assessed, how the quality of individual studies will be assessed, and whether and how this will influence the planned synthesis.. |
| 28 | Strategy for data synthesisGive the planned general approach to be used, for example whether the data to be used will be aggregate or at the level of individual participants, and whether a quantitative or narrative (descriptive) synthesis is planned. Where appropriate a brief outline of analytic approach should be given. |
| 29 | Analysis of subgroups or subsetsGive any planned exploration of subgroups or subsets within the review. ‘None planned’ is a valid response if no subgroup analyses are planned. |
| Review general information | |
| 30 | Type and method of reviewSelect the type of review and the review method from the drop down list. |
| 31 | LanguageSelect the language(s) in which the review is being written and will be made available, from the drop down list. Use the control key to select more than one language.Will a summary/abstract be made available in English? |
| 32 | CountrySelect the country in which the review is being carried out from the drop down list. For multi-national collaborations select all the countries involved. Use the control key to select more than one country. |
| 33 | Other registration detailsGive the name of any organisation where the systematic review title or protocol is registered together with any unique identification number assigned. If extracted data will be stored and made available through a repository such as the Systematic Review Data Repository (SRDR), details and a link should be included here. |
| 34 | Reference and/or URL for published protocolGive the citation for the published protocol, if there is one.Give the link to the published protocol, if there is one. This may be to an external site or to a protocol deposited with CRD in pdf format.I give permission for this file to be made publicly available |
| 35 | Dissemination plansGive brief details of plans for communicating essential messages from the review to the appropriate audiences.Do you intend to publish the review on completion? |
| 36 | KeywordsGive words or phrases that best describe the review. (One word per box, create a new box for each term) |
| 37 | Details of any existing review of the same topic by the same authorsGive details of earlier versions of the systematic review if an update of an existing review is being registered, including full bibliographic reference if possible. |
| 38 | Current review statusReview status should be updated when the review is completed and when it is published. |
| 39 | Any additional informationProvide any further information the review team consider relevant to the registration of the review. |

##

***Supplementary File 2. Extraction equations that can be used prior to analysis to get missed variables***

**Estimation of mean and SD**

When the included study had reported the mean/median, range, and the sample size, the mean and SD were estimated by following equations [1]:

| $\mathrm{mean}=\frac{\min+2\mathrm{median}+\max}{4}$ | when sample size ≤25 |
| --- | --- |
| mean= median | when sample size >25 |
| $\mathrm{SD}=\sqrt{\frac{1}{12}\left[ \frac{{(\min-2\mathrm{median}+\max)}^{2}}{4}+{(\max-\min)}^{2} \right]}$ | when sample size ≤15 |
| $\mathrm{SD}=\frac{\max-\min}{4}$ | when sample size >15-≤70 |
| $\mathrm{SD}=\frac{\max-\min}{6}$ | when sample size >70 |

**Estimation of unreported SD** [2]

When the study reported the mean only, linear (log( SD) vs log(mean)) chart was used, in which values were extracted from other included studies.^[1,2]^

log(unreported SD) = Log(reported mean)*a+b

References supplementary Table 3

[1] Hozo SP, Djulbegovic B, Hozo I (2005) Estimating the mean and variance from the median, range, and the size of a sample. BMC Med Res Methodol 5: 13.

[2] van Rijkom HM, Truin GJ, van 't Hof MA (1998) A meta-analysis of clinical studies on the caries-inhibiting effect of fluoride gel treatment. Caries Res 32: 83-92.

***Supplementary File 3. R codes and its guidance for meta-analysis done for comparison between EBOLA vaccine A and placebo***

***#Meta-analysis of Ebola vaccine safety***

**#1. Load meta package and xlsx package to load Excel table**

library(meta)

library(xlsx)

**#2. Load the data in Excel (note, naming the data is free, vaccinesafety.dat. You should not use "space" in R for the name)**

vaccinesafety.dat<-read.xlsx("D:\\Researcher\\S16\\imaginary data set.xlsx", sheetName="Safety") #The directory is depend on where you save the file (just right click the file and choose properties, copy paste the file location and make it similar to the format in this example)

vaccinesafety.dat

#Since many adverse events reported, we can do meta analysis to evaluate the odd of adverse event (each of them) and subgroup analysis

#First, do meta analysis of adverse event arthralgia by applying below codes

**#3. Load only arthralgia data**

arthralgia.dat<-vaccinesafety.dat[1:6,] #vaccinesafety.dat[1:6,] means load row 1 to 6, all columns

arthralgia.dat

**#4. Meta analysis of adverse events arthralgia (to learn about the code more, please check the guideline of meta package in the link provided at manuscript)**

OR.arthralgia = metabin (et, nt, ec, nc, studlab = Study, data = arthralgia.dat, method = "Inverse", sm = "OR")

print(summary(OR.arthralgia), digits = 2)

forest(OR.arthralgia, comb.fixed = FALSE,lab.e="Vaccine A", lab.c="Placebo", xlab="Odds of arthralgia", col.square = "green", col.diamond = "blue", print.Q = TRUE, print.pval.Q = TRUE)

funnel(OR.arthralgia)

metabias(k.min=6, OR.arthralgia)

#**5. The meta analysis above for arthralgia should be repeated for each of the adverse events. Start from loading the data of only specific adverse events desired (from step 3)**

**#Finally, subgroup analysis of adverse events related to Ebola vaccine A**

OR.adverseevents = metabin (et, nt, ec, nc, studlab = Study, data = vaccinesafety.dat, method = "Inverse", sm = "OR")

print(summary(OR.adverseevents), digits = 2)

**#6. Subgroup analysis**

analysis2<-update(OR.adverseevents, byvar=Adverse.events, print.byvar=FALSE)

print(summary(analysis2), digits=2)
